# Palatal Supernumerary Tooth in a Pediatric Patient

**DOI:** 10.7759/cureus.49751

**Published:** 2023-11-30

**Authors:** Douglas P Nanu, Nicole M Favre, Jeremy Walsh, Cameron Farsar, Tyler A Le, Michele M Carr

**Affiliations:** 1 Otolaryngology, Washington State University, Spokane, USA; 2 Otolaryngology, Jacobs School of Medicine and Biomedical Sciences, University at Buffalo, Buffalo, USA; 3 Emergency Medicine, Arrowhead Regional Medical Center, Colton, USA; 4 Education, American University of the Caribbean, Cupecoy, SXM

**Keywords:** extra tooth, congenital lesion, pediatric, lesion, supernumerary tooth

## Abstract

This report describes the case of a child with a congenital palatal lesion that grew rapidly in the first year of life and was found to be a supernumerary tooth. A 14-month-old male presented with a congenital midline palatal lesion visible behind his newly erupted maxillary central incisors. The lesion had been present since birth and was round, raised, firm, and covered with normal-appearing mucosa. The results from CT imaging indicated the lesion was a rudimentary tooth crown. It was excised and confirmed to be a supernumerary tooth. The patient healed without complications. Congenital palatal lesions with this appearance are most commonly hamartomas, cysts, epulides, and teratomas. Congenital midline palatal lesions are uncommon, and supernumerary teeth are not typically in the differential diagnosis. Imaging is helpful for the management of congenital palatal lesions.

## Introduction

Pediatric oral cavity diseases face misdiagnosis or inadequate treatment, primarily attributable to insufficient resources and lack of parental education [1]. Congenital oral cavity masses comprise a broad spectrum of diseases, with variable effects on airway and swallowing and differing amenabilities to treatment [2]. Supernumerary teeth are rarely a differential diagnosis for congenital palatal lesions. Previous articles addressing pediatric oral masses have consistently omitted consideration of these congenital lesions [2,3]. A supernumerary tooth is any tooth or tooth substance within the oral cavity that is in addition to the 20 deciduous or 32 permanent teeth [3]. The prevalence of supernumerary teeth in the United States is between 0.15% and 3.9%, with 80-90% occurring in the maxilla [4]. Here, we report a case of a child with a congenital palatal lesion that was found to be a supernumerary tooth.

## Case presentation

**Figure 1:**
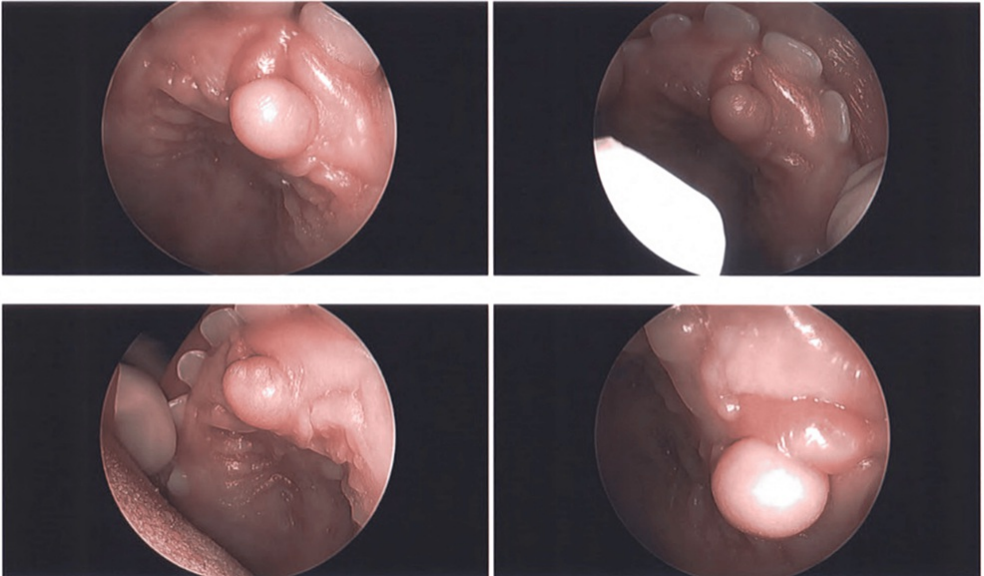
Firm, round, and slightly pedunculated lesion (4 mm) overlying the incisive papilla.

A 14-month-old male initially presented to our otolaryngology academic department for a congenital palatal lesion that had been increasing in size since birth. It was round, symmetrical, pink, and visible behind his maxillary central incisors. There was no history of oral trauma. He was able to eat without difficulty. Speech was developing appropriately, and his voice was normal. He was otherwise healthy apart from his parents’ concern that he was not hearing normally. On examination, he had bilateral dull and opaque tympanic membranes. Oral cavity examination revealed a firm, round, slightly pedunculated 4 mm lesion overlying the incisive papilla (Figure 1).

**Figure 2:**
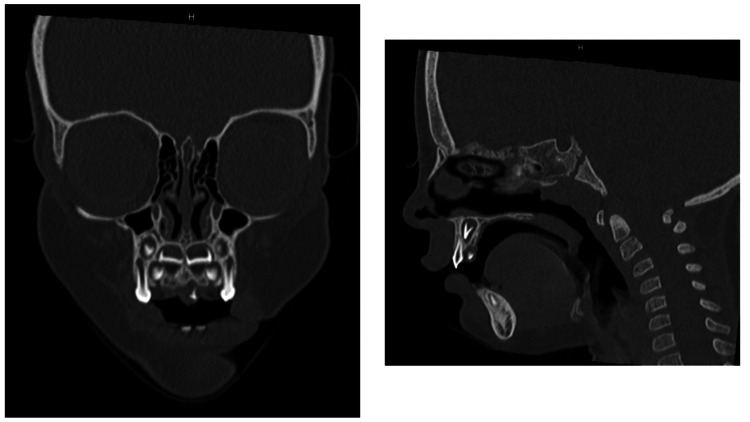
CT scan showing a radiopaque fusiform mass underlying the oral lesion with intact palatal bone.

Five months later, he presented with clear ears and normal audiometric testing, but the oral lesion had grown to 8 mm in diameter. Primary dentition was erupting normally, with four anterior normal-appearing maxillary teeth in situ. A CT scan showed a radiopaque fusiform mass underlying the oral lesion with intact palatal bone (Figure 2).

**Figure 3:**
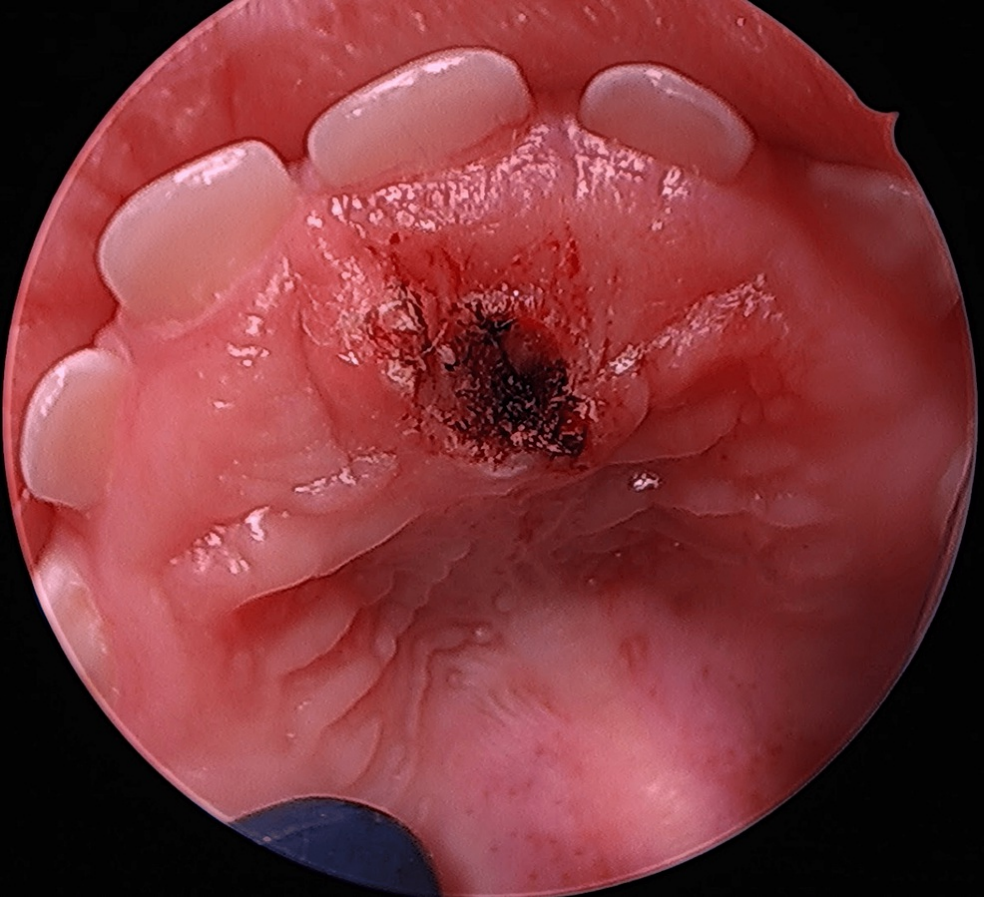
Post-operative wound of the supernumerary tooth mass excision via electrocautery.

At 21 months of age, the patient was placed under general anesthesia, and the mass was excised with electrocautery. The specimen appeared to contain a small hard white fusiform body closely adherent to normal mucosa. The wound was left to granulate. The patient tolerated the excision and the postoperative healing period well and continued eating and drinking normally. At a two-week follow-up, the palate appeared completely normal. Pathology revealed that the mass contained a supernumerary tooth with normal overlying mucosa (Figure 3). 

## Discussion

Congenital palatal lesions are a common concern among parents and healthcare providers because of their diverse etiologies. The differential diagnosis for these anomalies consists of palatal cysts, benign and malignant neoplasms, and embryological/congenital masses. These lesions can have severe effects on a child’s ability to breathe, speak, and feed. Therefore, early diagnosis and management of these conditions is critical to help ensure proper development.

Palatal cysts

Among the possible etiologies, palatal cysts are the most common, appearing in 65-85% of newborns [5]. Palatal cysts can be further subdivided into three subtypes: Bohn’s nodules, Epstein pearls, and eruption cysts (including eruption hematomas). Although both Bohn’s nodules and Epstein pearls contain keratin and are lined by parakeratinized stratified squamous epithelium, they differ in location. Bohn’s nodules, with a prevalence of 47.4% in newborns, appear at the junction of the hard and soft palate, whereas Epstein pearls, with a prevalence of 35.2% in newborns, typically appear as nodules in the mid-palatal raphe, along the line of fusion [6,7]. Treatment is generally unnecessary for either of these because they eventually disappear spontaneously. Eruption cysts are rare in newborns, appearing as bluish, dome-like translucent nodules overlying erupting teeth [6,7]. They are more common in children whose primary dentition is erupting. Eruption cysts also normally resolve with the eruption of the teeth. However, if the eruption cyst is symptomatic, it can be treated with simple surgical excision [8].

Benign/malignant neoplasms

Other palatal lesion etiologies include the broad category of benign and malignant neoplasms. One of the most common types of benign neoplasms of the palate is congenital or infantile hemangioma, which is formed as a result of abnormal proliferation of blood vessel cells. These lesions often involute spontaneously or with the use of beta blockers. Surgical management is considered if the lesion is symptomatic or persists [2]. Benign neoplasms of the palate can also be congenital lymphangiomas, also known as lymphatic malformations, which form in the lymphatic system during fetal development. Lymphangiomas often appear blue or red with hemorrhagic superficial vesicles and account for 25% of all benign pediatric vascular tumors [9]. The treatment of choice for lymphangiomas is a wide local excision of the lymphatic channel that is affected [9]. Mucoepidermoid carcinoma (MEC) is an epithelial salivary gland malignant neoplasm that can occur in the pediatric population. While these lesions usually arise in the parotid gland, about one-third of lesions develop in minor salivary glands [10]. Thus, lesions can arise anywhere in the oral cavity, including the palate. These lesions usually appear as firm and painless light blue masses, which mimic other vascular lesions, like a mucocele or hemangioma [10]. Therefore, imaging and fine needle aspiration are needed for a definitive diagnosis. Low to intermediate-grade MECs can be managed surgically, while high-grade MECs require surgery and postoperative chemotherapy and radiation [10].

Embryological and congenital lesions

Nasopalatine duct cysts (NPDCs) should also be on the differential as these cysts develop in the midline of the anterior maxilla, near the incisive foramen [11]. NPDCs arise from the embryological remnants that connect the anterior maxilla and nasal cavity [11]. NPDCs often present with pain, drainage, and tooth displacement [11]. While these lesions mainly occur in the fourth to sixth decades of life, they can arise at any time. Diagnosis is done through imaging and can be treated with surgical enucleation [11]. One must also consider congenital palatal lesions, like epulis (congenital granular cell tumor) or epignathus [2]. Epulis are extremely rare, affecting only six per one million neonates [2]. These lesions often arise from the gingiva mucosa or at the alveolar ridge of the maxilla or mandible and are confined to the gingiva in neonates [2]. Epulis presents as a single pedunculated gingival mass that has a tendency to bleed. While most lesions are incidentally found in utero by ultrasound, a definitive diagnosis is made histologically [2]. Treatment of epulis is done surgically, resulting in very good outcomes for patients [2].

There is also the possibility that a palatal lesion may be an epignathus, which is one of the rarest congenital palatal masses with an incidence of one per 35,000-200,000 live births [9]. Epignathus is a pharyngeal teratoma made up of all three germ cell layers but with a dominant ectodermal component. These pharyngeal teratomas attach at the base of the skull, typically at the palate, and grow into the oral cavity potentially causing an obstructed airway if they are too large [2].

The current case

In the present case, the congenital midline palatal lesion was a supernumerary tooth, or dental hyperdontia, which is not a common differential diagnosis. Supernumerary teeth can occur in any region of the dental arches and most commonly involve permanent teeth of the maxilla [3]. The palate is a very rare location for hyperdontia, and our report of an isolated supernumerary tooth in the palate of a pediatric patient is, to the best of our knowledge, the second reported case in the literature [12].

The etiology of supernumerary teeth, though not well defined, is theorized to be hyperactivity of dental lamina (the hyperactivity theory) [13]. An alternate theory is that a supernumerary tooth results from abnormal division of a tooth bud. These lesions commonly develop in conjunction with cleft lip or palate, cleidocranial dysplasia, Gardner syndrome, and trisomy 21. They commonly displace the crowns of the incisors but can displace other permanent teeth and can erupt ectopically. In addition, supernumerary teeth result in cyst formation in 30% of cases, with the potential to lead to the reabsorption of adjacent tooth roots [13].

Failure of tooth eruption, ectopic eruption, and the persistence of deciduous teeth are all factors that point toward hyperdontia [13]. Unerupted supernumerary teeth are commonly found as incidental findings on radiographic images [13]. Although a complete radiographic survey of the entire oral cavity is necessary to visualize these lesions, CT imaging is being used more regularly [14]. The management of supernumerary teeth begins with a thorough history, clinical examination, and appropriate diagnostic testing. Sometimes there is an advantage to preserving the tooth, which may require endodontic therapy, in which the tooth is devitalized, and frequent monitoring may be necessary [13]. Removal of the tooth is recommended when there is delayed tooth eruption, risk of caries, displacement, and/or severely rotated teeth. Supernumerary teeth may also need to be removed for orthodontic treatment, alveolar bone grafting, dental implant placement, or aesthetics and function [14]. Early excision prevents potential damage from crown and root growth and elongation of the tooth. Risks of removal include damage to the nerve and blood vessels during the manipulation of the tooth, perforation of the maxillary sinus, and disruption of structures in the pterygomaxillary space or orbit, depending on the tooth’s location [13]. 

## Conclusions

The management of the supernumerary tooth in the palate in the present case was simple because the tooth was parallel to the bony palate, with soft tissue separating it from the bone. Congenital midline palatal lesions are uncommon, and supernumerary teeth are not typically in the differential diagnosis. This case reminds us to keep this possibility in mind. Imaging is helpful in the management of congenital palatal lesions.
